# The Burden of Drug-Resistant Tuberculosis in Papua New Guinea: Results of a Large Population-Based Survey

**DOI:** 10.1371/journal.pone.0149806

**Published:** 2016-03-22

**Authors:** Paul Aia, Margaret Kal, Evelyn Lavu, Lucy N. John, Karen Johnson, Chris Coulter, Julia Ershova, Olga Tosas, Matteo Zignol, Shalala Ahmadova, Tauhid Islam

**Affiliations:** 1 National Department of Health, Port Moresby, Papua New Guinea; 2 Health & HIV Implementation Services Provider, Abt JTA, Port Moresby, Papua New Guinea; 3 Queensland Mycobacterium Reference Laboratory, Pathology Queensland, Australia; 4 Centers for Disease Control and Prevention (CDC), Atlanta, Georgia, United States of America; 5 Guy’s and St Thomas’ NHS Foundation Trust, London, United Kingdom; 6 Global Tuberculosis Programme, World Health Organization, Geneva, Switzerland; 7 Stop TB and Leprosy Elimination, World Health Organization, Western Pacific Regional Office, Manila, Philippines; 8 World Health Organization, Port Moresby, Papua New Guinea; St. Petersburg Pasteur Institute, RUSSIAN FEDERATION

## Abstract

**Background:**

Reliable estimates of the burden of multidrug-resistant tuberculosis (MDR-TB) are crucial for effective control and prevention of tuberculosis (TB). Papua New Guinea (PNG) is a high TB burden country with limited information on the magnitude of the MDR-TB problem.

**Methods:**

A cross-sectional study was conducted in four PNG provinces: Madang, Morobe, National Capital District and Western Province. Patient sputum samples were tested for rifampicin resistance by the Xpert MTB/RIF assay and those showing the presence of resistance underwent phenotypic susceptibility testing to first- and second-line anti-TB drugs including streptomycin, isoniazid, rifampicin, ethambutol, pyrazinamide, ofloxacin, amikacin, kanamycin and capreomycin.

**Results:**

Among 1,182 TB patients enrolled in the study, MDR-TB was detected in 20 new (2.7%; 95% confidence intervals [CI] 1.1–4.3%) and 24 previously treated (19.1%; 95%CI: 8.5–29.8%) TB cases. No case of extensively drug-resistant TB (XDR-TB) was detected. Thirty percent (6/20) of new and 33.3% (8/24) of previously treated cases with MDR-TB were detected in a single cluster in Western Province.

**Conclusion:**

In PNG the proportion of MDR-TB in new cases is slightly lower than the regional average of 4.4% (95%CI: 2.6–6.3%). A large proportion of MDR-TB cases were identified from a single hospital in Western Province, suggesting that the prevalence of MDR-TB across the country is heterogeneous. Future surveys should further explore this finding. The survey also helped strengthening the use of smear microscopy and Xpert MTB/RIF testing as diagnostic tools for TB in the country.

## Introduction

Tuberculosis (TB) remains a major challenge to public health worldwide. In 2014, the World Health Organization (WHO) estimated that approximately 9.6 million people developed TB globally and about 480,000 were infected with multidrug-resistant TB (MDR-TB) strains. MDR-TB is a form of TB with in-vitro resistance to the two most potent anti-TB drugs, isoniazid, and rifampicin, with or without resistance to any other drugs [1]. Inadequate use of anti-TB drugs favours the emergence of drug resistance, which may then be transmitted to others. MDR-TB patients require a treatment which commonly lasts at least 20 months and employs drugs that are difficult to procure and more toxic and expensive than those used to treat drug-susceptible TB [2,3,4].

Resistance to anti-TB drugs is considered an emerging problem in Papua New Guinea (PNG), but a reliable estimate of the prevalence of MDR-TB in the country is not available. Studies performed by the Queensland Mycobacterium Reference Laboratory (QMRL) on isolates from patients of the South Fly District (Western Province) seeking cross border care in Australia suggest high rates of MDR-TB: Gilpin *et al*.[5] reported 25% of MDR-TB in new cases and Simpson *et al*. [6] reported a similar finding (26%). Data from the PNG Institute of Medical Research (Ballif, Harino *et al*.) [7] reported 5.2% of MDR-TB in a cohort of TB patients from Madang PNG, the majority of whom were new cases. A study from Kikori in Gulf Province of PNG reported 9% (3 of 32 TB cases) of rifampicin resistant cases among TB patients (Cross, Coles *et al*.) [8] and another one reported MDR-TB in 2.8% of all cases tested in three sites (Ley, Harino et al) [9]. These studies were small in nature and not designed to obtain population-representative data, therefore a large representative survey was needed to determine the real burden of anti-TB drug resistance in PNG.

The survey aimed to provide to the national TB control programme (NTP) and its partners reliable information on the prevalence and risk factors for MDR-TB in the country to better target prevention and control efforts. The findings of this study are relevant beyond the country’s borders as these add to the current knowledge about the burden of MDR-TB in the region.

## Materials and Methods

### Study Design and Sampling Method

The survey was conducted by the NTP. Cluster-randomised sampling was used to obtain a representative sample of newly registered smear-positive pulmonary TB patients identified by public healthcare services in Madang, Morobe, Western Provinces and National Capital District. For logistic and feasibility reasons, the number of clusters to be included in the survey was set to 40, with a target cluster size of 24 new sputum-smear positive patients in 17 out of 19 districts of the selected provinces. The 40 clusters were distributed in 27 health centers selected using a probability-proportional to size cluster sampling strategy. As often recommended in these surveys, no separate sample size was calculated for previously treated cases.[10]

The sample size for new TB cases as defined above was set at 978. Sample size calculation was based on the 2010 estimated number of new smear positive TB cases in the area (n = 2,747) and assumed 5% expected prevalence of MDR-TB among newly diagnosed patients and 20% expected sample losses due to insufficient sputum volume, culture contamination and/or susceptibility tests not interpretable. The precision for the 95% confidence interval was set at ± 2% and the design effect at 2, effectively doubling the required sample size, to account for heterogeneity in resistance between clusters.

### Recruitment Period

The study took place from October 2012 to December 2014. During this period, patients presenting to any of the survey sites with features of pulmonary TB underwent clinical and sputum examination. Patients with sputum smear-positive TB who provided informed consent were enrolled in the study. Children under the age of 15 who satisfied the definition of a smear-positive TB case were also eligible for the study. All patients were offered HIV testing and counselling. Specimens were also collected from all previously treated cases registered in the selected diagnostic centres during the intake period of new smear-positive patients. A questionnaire to access history of previous treatment for TB was administered to all patients enrolled as per WHO recommendations [10].

### Laboratory Procedures

Two sputum samples were collected from each patient and transported to the appointed/nearest sputum microscopy centre. On arrival both samples were registered in the laboratory register. Two smears were made and examined by Ziehl–Neelsen stain as per current NTP guidelines. If either specimen was smear-positive, both samples were referred to the designated laboratories (provincial laboratories or central public health laboratory) to undergo testing with Xpert MTB/RIF (Cepheid, Sunnyvale, CA, USA). In the circumstance that both TB and rifampicin resistance was detected, the second sample was sent to the QMRL, the supra national TB reference laboratory (SRL), for culture and drug sensitivity testing (DST).

At the SRL, sputum samples were digested and decontaminated with 4% NaOH followed by neutralisation and centrifugation. Sediment was subjected to smear and culture. An aliquot was inoculated onto an Löwenstein-Jensen slope containing glycerol, incubated at 37°C and also into a Mycobacteria Growth Indicator Tube (MGIT) with PANTA following the manufactures instructions and incubated in the BACTEC^TM^ MGIT^TM^ 960 instrument (Becton Dickinson, Sparks, MD, USA). Positive cultures were examined by smear and *Mycobacterium tuberculosis* confirmed by SD Bioline TBAgMPT64 rapid assay (Standard Diagnostics, Inc., Gyeonggi-do, Republic of Korea). Non-tuberculous mycobacteria were identified to species level. First and second line DST was performed according to the manufacturers instructions in the BACTEC^TM^ MGIT^TM^ 960 system. Critical concentrations were as follows: rifampicin 1.0 mg/L, isoniazid 0.1 / 0.4 mg/L, ethambutol 5.0 mg/L, streptomycin 1.0 mg/L, pyrazinamide 100 mg/L, ofloxacin 2.0 mg/L, amikacin 1.0 mg/L, capreomycin 2.5 mg/L and kanamycin 2.5mg/L.

### Data Management and Statistical Analysis

A standardized questionnaire was used by trained health workers in each of the 27 selected health centers to collect demographic and clinical information, including TB treatment history and HIV status. All data collected in the questionnaires were entered into an Epi Info based database by the provincial survey coordinators. Results from smear microscopy, XpertMTB/RIF testing, culture and DST were reviewed and also entered into the same database. MDR-TB prevalence estimates among new and previously treated patients were obtained by fitting logistic regressions with adjusted (robust) standard errors to account for clustering on the primary sampling unit. In addition, given that the sampling design requires that an equal number of new cases is available in each cluster, sample weights to account for over/under- enrolment by cluster were included in the regression models. The analysis was performed following multiple imputation of missing observations based on a probability model of the complete data for age, gender, treatment history, rifampicin, isoniazid and MDR. Associations between MDR-TB and patients’ age, gender and history of previous anti-TB treatment were explored in a multiple logistic regression analysis which accounted for the cluster design and variation in cluster size as described earlier. Only cases with complete data were included in the analysis of risk factors. Measures of association for potential predictors were summarised by odds ratios and the corresponding 95% confidence intervals. Summary statistics and statistical analyses were conducted in STATA SE/12.1 (StataCorp LP, TX, USA).

### Ethical Clearance

The survey protocol, developed according to international recommendations [10], was approved by the Medical Research Advisory Committee of the Ministry of Health. All patients were asked to sign a consent from before enrolment in the survey. Written consent was obtained from the guardians of children enrolled in the study. Confidentiality was respected throughout data collection, management and analysis. Unique identifiers were assigned and patient identifiers (e.g. name, surname, address) were not captured in the database. Ownership of survey data remains with the NTP.

## Results

**Fig 1:**
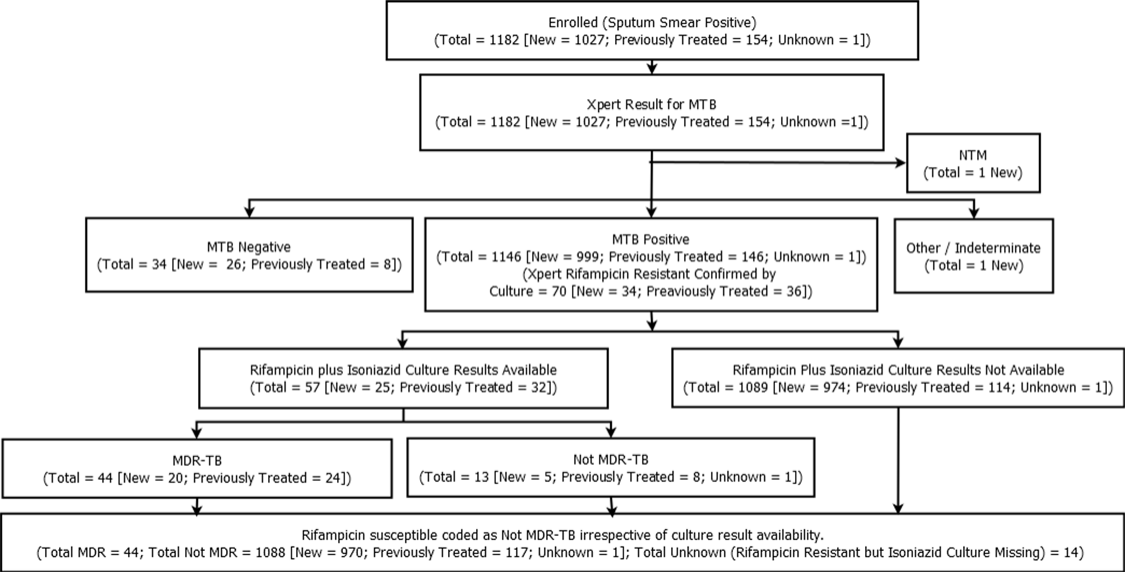
Flow chart of enrolled patients.

During the intake period of the survey, 1,182 patients with sputum-smear positive pulmonary TB were enrolled. No patient was excluded because of refusal to participate. Of them, 1,027 were newly diagnosed cases, 154 patients had previous history of TB treatment and for one patient, information on history of treatment could not be retrieved. All enrolled cases underwent Xpert MTB/RIF testing. A total of 1,146 patients were detected with TB (999 new cases, 146 previously treated cases and 1 case with undocumented history). [Fig 1]

Of the 1,146 patients with TB, 590 (51.0%) were male. The median age was 28 years (interquartile range [IQR]: 21–37).Among males the median age was 30 years (IQR: 21–39) and among females the median age was 27 years (IQR: 21–35).

Information on HIV status was available for 651 of all cases with TB (56.81%). Among them 32 (4.92%) cases were HIV positive.

Table 1 shows the distribution of resistance to rifampicin and isoniazid in new and previously treated patients. Resistance to rifampicin was observed in 70/1146 (6.1%) cases (3.4% of new cases and 25.3% of previously-treated). All rifampicin-resistant cases were intended to undergo culture and DST at the SRL, however culture and DST results were available for 57 of them due to non-growth of 11 samples and loss of 3 samples during transport. Of the 57 cases with culture and DST result, 44 (77.2%) cases had additional resistance to isoniazid. Of the 44 MDR-TB cases 20 were in new and 24 were in previously treated TB cases.

**Table 1:** Proportions of resistance to rifampicin isoniazid and MDR in new and previously treated TB cases in 4 provinces in PNG from Oct 2012 to Dec 2014.

	New		Previously Treated	
	n (N = 999)	%	n (N = 146)	%
**Rifampicin**	34	3.4 (2.4–4.7)	36	24.66 (17.9–32.5)
**Isoniazid**	20	2.02 (1.2–3.1)	24	17.02 (11.2–24.3)
**MDR**	20	2.02 (1.2–3.1)	24	17.02 (11.2–24.3)

Table 2 shows the DST results of anti TB drugs other than rifampicin and isoniazid. No XDR-TB was detected. Resistance to ofloxacin was detected in one (1) case (1.8%) and resistance to any of the second-line injectable drugs (kanamycin, amikacin, capreomycin) was found in 3 cases (5.3%).

**Table 2:** Proportions of resistance to second-line anti-TB drugs among patients with rifampicin resistances in 4 provinces in PNG from Oct 2012 to Dec 2014.

	Rifampicin Resistant		MDR-TB	
	N (N = 57)	%	n(N = 44)	%
Ethambutol	14	25.0 (14.4–38.4)	14	32.6 (19.1–48.5)
Streptomycin	31	54.4 (40.7–67.6)	29	65.9 (50.1–79.5)
Kanamycin	1	1.8 (0.1–9.6)	1	2.3 (0.1–12.0)
Amikacin	1	1.8 (0–9.4)	1	2.3 (0.1–12.0)
Capreomycin	3	5.3 (1.1–14.6)	1	2.3 (0.1–12.0)
Ofloxacin	1	1.75 (0–9.4)	1	2.3 (0.1–12.0)
Pyrazinamide	16	28.1 (17.0–41.5)	16	36.4 (22.4–52.2)

Overall, after imputation of missing observations, proportions of MDR-TB were 2.7% (95%CI:1.1–4.3%) in new and 19.1% (95%CI:8.5–29.8%) in previously treated TB cases. Rifampicin resistance was found in 3.4% (95%CI:1.7–5.0%) of new and 25.6% (95%CI:14.9–36.3%) of previously treated TB cases.

Table 3 shows the distribution of MDR-TB cases across diagnostic centres. One centre, Daru Hospital in Western Province, shows higher percentage (34.2%) of MDR-TB. Also 30% (6/20) of all new and 33.3% (8/24) of all previously treated cases identified with MDR-TB during the survey were from that hospital.

**Table 3:** Distribution of MDR Cases across Diagnostic Centres (where at least 1 MDR-TB case was found).

		MDR			
	New Cases	Previously treated Cases	Total	MTB Patients Enrolled	Percentage MDR
6 Mile/ NCD	1	2	3	140	2.1
9 Mile/NCD	1	1	2	41	4.9
Angau/Morobe	3	1	4	30	13.3
Badili/NCD	2	1	3	31	9.7
Boana/Morobe	0	1	1	32	3.1
Bogia/Madang	0	2	2	27	7.4
Daru_Hospital/WP	6	8	14	41	34.2
Gerehu/NCD	1	3	4	125	3.2
Kilakila/NCD	0	1	1	56	1.8
Lawes_Road/NCD	2	1	3	54	5.6
Modilon/Madang	1	1	2	96	2.1
Mugil/Madang	0	1	1	25	4.0
Port Moresby General Hospital/NCD	1	0	1	57	1.8
Tokarara/NCD	2	1	3	40	7.5

(NCD: National Capital District, WP: Western Province)

The probability of MDR-TB was eleven fold higher in patients previously treated with anti-TB drugs as compared in newly registered cases (OR 11.3 (6.4–19.8), P<0.001). (Table 4).

**Table 4:** Risk factors for MDR-TB Patients.

Variable	Level	Odds Ratio	p-value	95% Confidence Intervals
**Gender** (Reference: Male)	Female	0.9	0.75	(0.4–1.9)
**Treatment History** (Reference: New)	Previously treated	11.2	< .0001	(6.4–19.8)
**Age Group** (Reference: 25–34 years)	0–24 years	1.3	0.48	(0.6–2.7)
	35–44 years	2.0	0.10	(0.9–4.7)
	45–54 years	0.3	0.25	(0.1–2.2)
	55–68 years	0.4	0.29	(0.1–2.3)

The analysis included only complete cases (n = 1,130 cases; 40 clusters), defined as those where MDR status, gender, treatment history and age were available.

## Discussion

This study represents the first large population-based survey to describe the levels of anti-TB drug resistance in PNG. As per census data the four provinces included in the survey (Madang, Morobe, NCD and Western) account for 24% (1,734,192/7,275,324) of the country population [11]. However, more than 50% of the new smear positive TB cases reported nationwide in 2010 were notified in these provinces.

The levels of MDR-TB found in PNG are higher than those reported by neighboring countries such as Indonesia (1.9% in new and 12% in previously treated TB cases) and Australia (1.7% in new and 10% in previously treated TB cases) and similar to high MDR-TB burden countries in Pacific region, such as the Philippines (2.0% in new and 21% in previously treated TB cases) and Viet Nam (4.0% in new and 23% in previously treated TB cases).[1,12]

In our study most (93%) of the MDR-TB cases were individuals in the productive age, 15–45 years, with a male to female ratio of 1:1.1. It is noteworthy that 45% of MDR-TB cases detected in this survey were not previously exposed to anti-TB drugs. Drug-resistance in cases not previously exposed to anti-TB drugs generally indicates primary infection with resistant strains and points to shortfalls in infection control measures and is an indicator of late diagnosis.

In Daru Hospital in Western Province a higher proportion of drug resistance was detected. Although the survey was not designed to estimate prevalence of drug resistance separately in the different provinces, this finding suggests high transmission of MDR-TB in this setting and requires further investigation. In the same setting the number of notified TB patients has been increasing over the past few years (59 cases in 2012, 61 in 2013 and 84 in 2014) [13]. The higher prevalence of drug resistance in this area could be explained by long history of poor supervision of treatment, large proportion of patients who don’t finish the treatment course, poor quality diagnosis (with smear microscopy) or limited capacity to promptly identify patients with drug resistance until recently. Overcrowded settlements and extreme poverty may also play a major role in fuelling this epidemic. Conducting ad hoc survey or even better establishing a continuous surveillance system in Western Province would be a priority to confirm this finding and monitor time trends in drug resistance.

Approximately one fourth of rifampicin resistant TB cases were resistant to ethambutol (14/56) and pyrazinamide (16/57) and therefore these drugs can still be beneficial to some rifampicin resistant TB patients when added to a second-line drug regimen. Representative surveillance data from a number of settings in the world indicate that on average 9.7% of MDR-TB cases (95%CI: 7.4–12.1) have XDR-TB(1). Although XDR-TB has been reported from PNG, in our survey we didn’t find any case [1].

Previous treatment history was significantly associated with MDR-TB after adjustment for age and gender. Though this is a well known findings globally it gives additional confidence to the NTP on the validity of the current national policy for MDR-TB screening. [13]

The country has a rugged and mountainous topography, road networks are limited and transport is a major issue. Only 3% of the roads are paved and many villages can only be reached by foot. Access to widely scattered rural communities is often difficult, slow and expensive. However, this study has proved that effective samples transport mechanisms can be established even in this difficult geographic context.

The use of a rapid diagnostic technique to screen rifampicin resistant cases in a representative drug resistance survey is novel and has proved to work well. It has substantially decreased the costs and need for in-country laboratory capacity. The survey has also served to strengthen capacity for sample collection and specimen transportation. The use of Xpert MTB/RIF assay for detection of TB and rifampicin resistance which has been piloted with this survey is now being expanded and will gradually improve access to TB diagnosis and care in the country. All rifampicin-resistant TB cases identified through the study were offered a standardised second-line treatment regimen as per national guidelines. The regimen was then modified according to the resistance profile detected by the DST performed at SRL. The survey showed that it is possible to set up screening mechanisms to identify drug resistance at the time of the TB diagnosis giving patients proper treatment at earlier stage of the disease.

Our study has several limitations. Firstly, in 2010, when the survey was designed, smear microscopy was not routinely performed and only 50% of newly registered TB patients across the four provinces had a smear microscopy result reported. Although this lack of information was factored into the sample size calculation it may have led to over or under-estimation of the survey sample size. Secondarily, a small proportion of smear-positive patients identified during the enrolment period were not enrolled in the study because they never came back to the site to collect the test results and enrol in the treatment programme.

This large population based survey represents the first attempt to describe and quantify the burden of resistance to anti-TB drugs in PNG. Levels of resistance are in line with those detected in other high TB burden countries of the region. The high proportion of MDR-TB found in Western Province was unexpected and suggests that the prevalence of MDR-TB across the country may be very heterogeneous. This finding should be further explored and a national survey should be conducted to have a better understanding of the distribution of drug-drug resistant TB in the country.

### Disclaimer

Authors alone are responsible for the views expressed in this publication and they do not necessarily represent the decisions or policies of their organizations.

## Supporting Information

S1 File Minimal data set. (CSV)
